# Genetic Determinants of Hydrogen Sulfide Biosynthesis in Fusobacterium nucleatum Are Required for Bacterial Fitness, Antibiotic Sensitivity, and Virulence

**DOI:** 10.1128/mbio.01936-22

**Published:** 2022-09-08

**Authors:** Yi-Wei Chen, Martha I. Camacho, Yimin Chen, Aadil H. Bhat, Chungyu Chang, Emily A. Peluso, Chenggang Wu, Asis Das, Hung Ton-That

**Affiliations:** a Division of Oral Biology and Medicine, School of Dentistry, University of California, Los Angeles, Los Angeles, California, USA; b Molecular Biology Institute, University of California, Los Angeles, Los Angeles, California, USA; c Department of Microbiology and Molecular Genetics, University of Texas Health Science Center, Houston, Texas, USA; d Department of Medicine, Neag Comprehensive Cancer Center, University of Connecticut Health Center, Farmington, Connecticut, USA; e Department of Microbiology, Immunology and Molecular Genetics, University of California, Los Angeles, Los Angeles, California, USA; KUMC

**Keywords:** *Fusobacterium nucleatum*, hydrogen sulfide, lanthionine, metabolism, virulence, preterm birth, placenta, electron microscopy

## Abstract

The Gram-negative anaerobe Fusobacterium nucleatum is a major producer of hydrogen sulfide (H_2_S), a volatile sulfur compound that causes halitosis. Here, we dissected the genetic determinants of H_2_S production and its role in bacterial fitness and virulence in this important member of the oral microbiome. F. nucleatum possesses four enzymes, CysK1, CysK2, Hly, and MegL, that presumably metabolize l-cysteine to H_2_S, and CysK1 was previously shown to account for most H_2_S production *in vitro*, based on correlations of enzymatic activities with gene expression at mid-log phase. Our molecular studies showed that *cysK1* and *megL* were highly expressed at the late exponential growth phase, concomitant with high-level H_2_S production, while the expression levels of the other genes remained substantially lower during all growth phases. Although the genetic deletion of *cysK1* without supplementation with a CysK1-catalyzed product, lanthionine, caused cell death, the conditional Δ*cysK1* mutant and a mutant lacking *hly* were highly proficient in H_2_S production. In contrast, a mutant devoid of *megL* showed drastically reduced H_2_S production, and a *cysK2* mutant showed only minor deficiencies. Intriguingly, the exposure of these mutants to various antibiotics revealed that only the *megL* mutant displayed altered susceptibility compared to the parental strain: partial sensitivity to nalidixic acid and resistance to kanamycin. Most significantly, the *megL* mutant was attenuated in virulence in a mouse model of preterm birth, with considerable defects in the spread to amniotic fluid and the colonization of the placenta and fetus. Evidently, the l-methionine γ-lyase MegL is a major H_2_S-producing enzyme in fusobacterial cells that significantly contributes to fusobacterial virulence and antibiotic susceptibility.

## INTRODUCTION

Hydrogen sulfide (H_2_S) is a volatile gas with a foul odor of rotten eggs that is enzymatically produced by both eukaryotic and prokaryotic cells. Considered a signaling molecule, H_2_S modulates many cellular processes, including cell proliferation, apoptosis, inflammation, and hypoxia (1–7). Mammalian cells produce H_2_S using three enzymes, cystathionine β-synthase, cystathionine γ-lyase, and 3-mercaptopyruvate sulfurtransferase, homologs of which are found in certain bacteria (8, 9). Studies of several aerobic bacterial species demonstrated that the deletion or inactivation of one or more of these enzymes results in the increased sensitivity of these bacteria to a wide range of antibiotics, and H_2_S-mediated antibiotic resistance likely occurs by the alleviation of oxidative stress imposed by antibiotics (8). Interestingly, H_2_S is produced by many anaerobic colonizers of the human oral cavity, such as Fusobacterium nucleatum, Parvimonas micra, and Porphyromonas gingivalis, and is associated with oral malodor or halitosis (10, 11).

**FIG 1:**
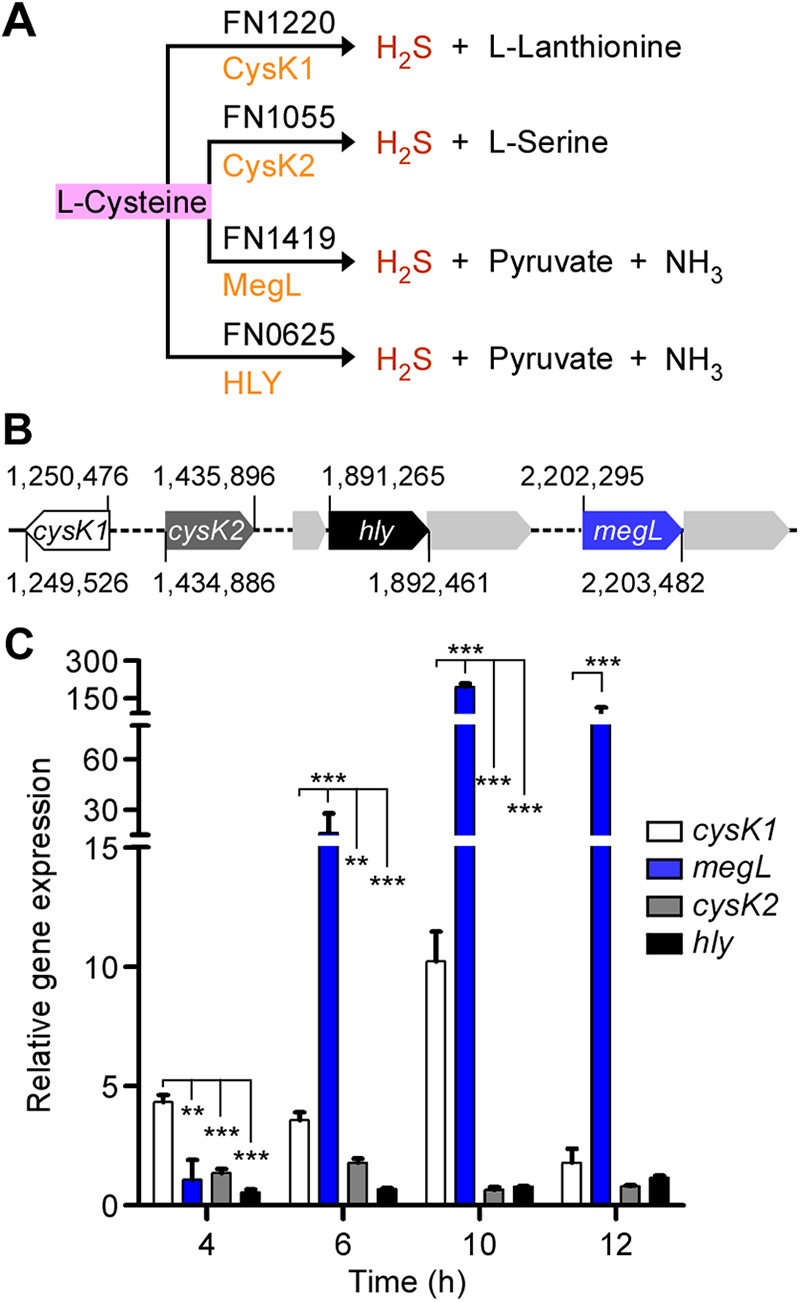
Enzymes involved in cysteine metabolism and gene expression in F. nucleatum. (A) In F. nucleatum strain ATCC 25586, FN1220, FN1055, FN1419, and FN0625 are enzymes involved in cysteine metabolism resulting in H_2_S production. Equivalent enzymes (orange) are predicted in strain ATCC 23726. (B) Locations of *cysK1*, *cysK2*, *hly*, and *megL* in the chromosome of F. nucleatum ATCC 23726. (C) Cultures of various F. nucleatum strains grown overnight were used to inoculate fresh cultures in Columbia broth with a starting OD_600_ of ~0.1. At timed intervals, the total RNA of each strain was isolated, and the expression levels of four cysteine metabolic genes, relative to the 16S rRNA gene, were determined by qRT-PCR. The results presented are averages from three independent experiments performed in triplicates. Statistical significance was analyzed by a *t* test (*, *P* < 0.05; **, *P* < 0.01; ***, *P* < 0.001).

Considered a major H_2_S-producing oral bacterium (10, 11), F. nucleatum is a prominent member of the oral microbiome. It not only contributes significantly to dental plaque formation through its ability to adhere to and interact with many distinct microbial species (12) but also can spread to extraoral sites such as the colon and placenta, promoting colorectal cancer and preterm birth (13, 14). Previous studies have identified four major enzymes involved in H_2_S production via cysteine metabolism in F. nucleatum (15–18). In the F. nucleatum ATCC 25586 strain, *FN0625* encodes an l-cysteine desulfhydrase (Fig. 1A) that catalyzes the α,β-elimination of l-cysteine to produce H_2_S, pyruvate, and ammonia (15). FN1220, annotated as cysteine synthase (https://biocyc.org), catalyzes the β-replacement of l-cysteine to produce H_2_S and lanthionine (17). Critically, lanthionine is part of the cell wall stem peptide that cross-links with a neighboring stem peptide during peptidoglycan assembly (19). *FN1055* encodes another predicted cysteine synthase that catalyzes the β-elimination of l-cysteine to produce H_2_S and l-serine (16). The fourth enzyme encoded by *FN1419*, annotated as l-methionine γ-lyase (MegL), is thought to catalyze the α,β-elimination of l-cysteine to produce H_2_S, pyruvate, and ammonia (18). Based on enzymatic activities *in vitro* and the relative mRNA levels of the four genes in ATCC 25596 cells determined at early log phase, FN1220 is estimated to account for the majority of H_2_S production (87.6%), with FN1055, FN1419, and FN0625 making up 10.3%, 1.94%, and 0.14% of the total H_2_S production, respectively (18). It remains unknown, however, whether one of these enzymes constitutes a major H_2_S production pathway *in vivo* or not and whether and how these enzymes are involved in fusobacterial physiology and virulence.

With recent advances in the fusobacterial genetics and genetic tractability of F. nucleatum ATCC 23726 (20–23), we sought to investigate the genetics of H_2_S production in F. nucleatum and its physiological regulation and function. We show here that the l-methionine γ-lyase MegL, encoded by the *FN1419* homolog, is a major H_2_S-producing enzyme *in vivo*. Furthermore, we show that MegL plays a significant role in fusobacterial virulence and antibiotic resistance since a mutant lacking *megL* is attenuated in a mouse model of preterm birth and exhibits increased tolerance to kanamycin (Kan) and decreased resistance to nalidixic acid (Nal). Finally, although the cysteine synthase CysK1, encoded by *FN1220*, marginally contributes to H_2_S production in cells, it is essential for cell viability and normal morphology due to its involvement in the generation of lanthionine, a key constituent of fusobacterial peptidoglycan.

## RESULTS

### Expression of hydrogen sulfide-associated genes in F. nucleatum.

To investigate the genetics and physiology of H_2_S production in fusobacteria, we chose F. nucleatum strain ATCC 23726, a urogenital isolate whose complete genome sequence is known and is amenable to markerless gene deletion and transposon mutagenesis (20, 21). As mentioned above, bioinformatics analysis of the ATCC 23726 genome revealed four genes predicted to encode homologs of H_2_S-producing enzymes corresponding to the *FN1220*, *FN1055*, *FN1419*, and *FN0625* genes (Fig. 1A and B). Two of these genes (*FN1220* and *FN1055*) encode predicted cysteine synthase A or CysK enzymes, which we renamed CysK1 and CysK2, respectively (Fig. 1A). *FN1419* is named *megL*, coding for l-methionine γ-lyase, whereas *FN0625* has been annotated as *hly* in strain ATCC 23726, coding for a pyridoxal phosphate-dependent aminotransferase (https://biocyc.org) (Fig. 1A).

**FIG 2:**
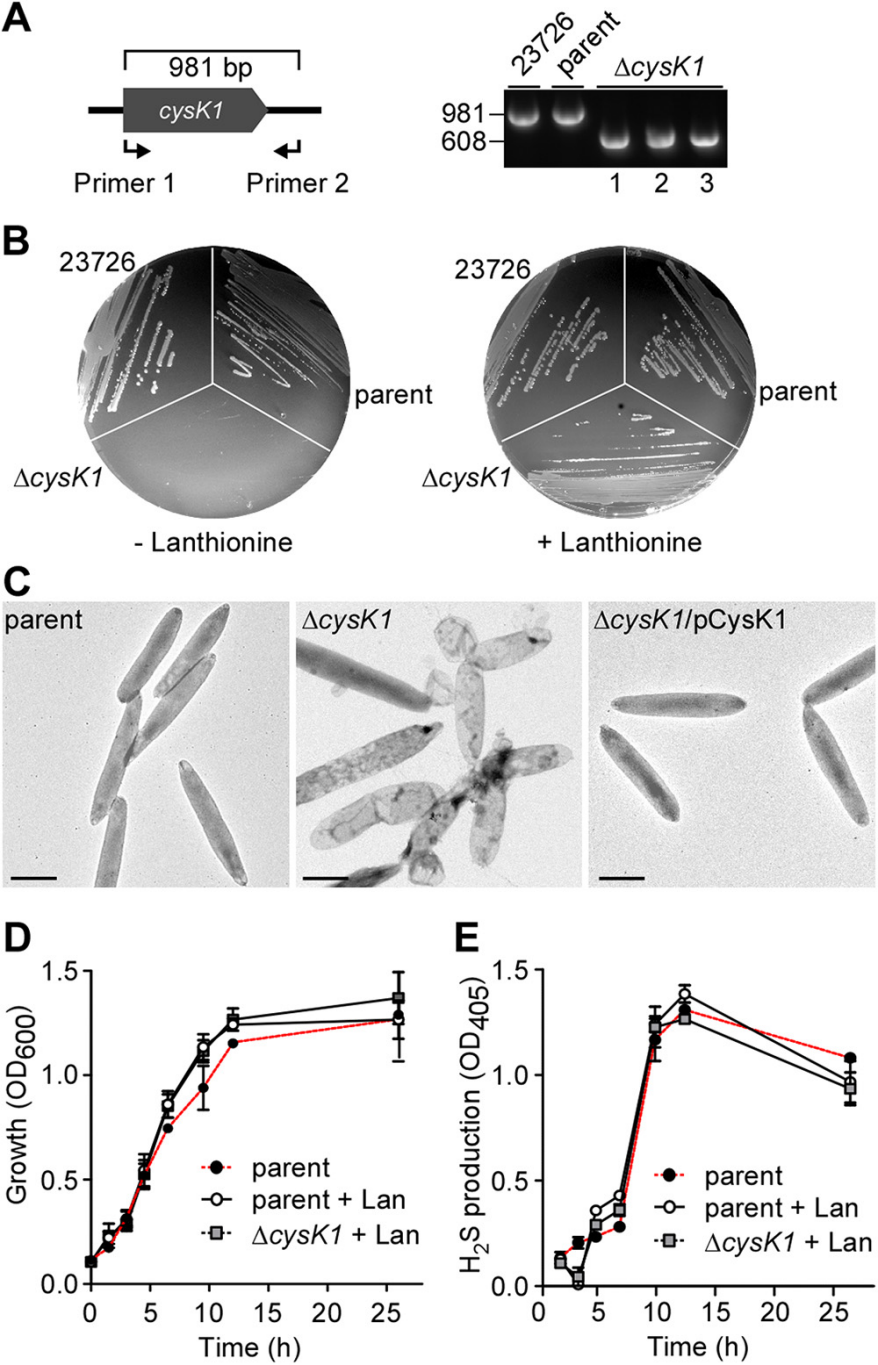
CysK1 is critical for cell survival of F. nucleatum. (A) A conditional *cysK1* deletion mutant was generated with a set of primers used to distinguish mutant alleles (608 nucleotides) from the ATCC 23726 strain (981 nucleotides) or its derivative CW1 (parent) by PCR amplification. (B) The ATCC 23726, parent, and conditional *cysK1* mutant (Δ*cysK1*) strains were streaked onto TSPC agar plates supplemented with or without l-lanthionine. (C) Cells of the indicted F. nucleatum strains grown without l-lanthionine for 3 h were harvested, immobilized on carbon-coated nickel grids, and stained with 1% uranyl acetate prior to observation using an electron microscope. Bars, 0.75 μm. (D) The growth of the indicated F. nucleatum strains in Columbia broth supplemented with l-lanthionine (Lan), or without (parent strain), was monitored by the OD_600_. (E) At timed intervals, aliquots were taken to determine H_2_S production levels using the bismuth method with the absorbance measured at 405 nm.

First, to examine the relative expression levels of each of these genes, the ATCC 23726 strain was cultured anaerobically in Columbia broth (Fisher), which contained 0.01% cysteine, and cells were harvested at different time points to isolate total RNA. Using specific oligonucleotide primers for the above-mentioned genes together with primers for 16S rRNA as a control, we then performed quantitative reverse transcription-PCR (qRT-PCR). Interestingly, *megL* was most highly expressed among the four genes, and the *megL* expression level was highest when the culture reached late log phase, with >100-fold induction of the *megL* transcripts in the early stationary phase (Fig. 1C; see Fig. 2D for growth curves). The expression level of *cysK1* also increased at late log phase, although it was approximately 15-fold lower than that of *megL* (Fig. 1C). In comparison, the expression levels of *cysK2* and *hly* were negligible, and the transcripts for these genes remained relatively unchanged over the time of culture (Fig. 1C). These results suggest that MegL and CysK1 play important roles in H_2_S production and function in F. nucleatum ATCC 23726 *in vivo*.

### CysK1 is essential for lanthionine biosynthesis and fusobacterial viability but dispensable for H_2_S production *in vivo*.

Previous studies demonstrated that FN1220 (or CysK1 in ATCC 23726) possesses the highest level of enzymatic activity for H_2_S production *in vitro* compared to FN1055 (CysK2), FN1419 (MegL), and FN0625 (Hly) (18). To examine how this relates to physiology *in vivo*, we sought to generate a deletion mutant of *cysK1* according to our previously reported protocols (20, 21), whereby the precise removal of chromosomal *cysK1* is achieved by two-step homologous recombination. First, an in-frame deletion construct in a plasmid containing 1-kb flanking DNA sequences upstream and downstream of *cysK1* was integrated into the fusobacterial chromosome by homologous recombination. Second, the excision of the plasmid sequence from the resultant strain by a subsequent homologous recombination event was selected for either the intact chromosomal *cysK1* allele or the deletion mutant allele that would be excised. However, we did not observe the expected result: the screening of many recombinants showed that none contained the desired deletion mutation after several attempts, as they all yielded only the wild-type allele. This is what is predicted if *cysK1* were an essential gene. Because lanthionine, an essential amino acid in the formation of fusobacterial peptidoglycan (24, 25), is a product of the CysK1-catalyzed reaction that produces H_2_S (Fig. 1A), we reasoned that supplementation with lanthionine might compensate for the loss of *cysK1* in fusobacteria and restore the viability of the Δ*cysK1* mutant. Indeed, by adding lanthionine to the selective medium in the second step of genetic manipulation mentioned above, we succeeded in obtaining the *cysK1* deletion mutant strain, as revealed by the PCR analysis (Fig. 2A).

To confirm that *cysK1* is essential for bacterial viability, the Δ*cysK1* mutant was restreaked onto agar plates with or without 1 mM lanthionine. As expected, while the Δ*cysK1* mutant was able to form colonies in the presence of lanthionine at a level comparable to that of the parent strain, it failed to form any colony in the absence of lanthionine (Fig. 2B). We next compared the cell morphologies of the parent, *cysK1* mutant, and *cysK1*-rescued strains by transmission electron microscopy (TEM), whereby harvested bacterial cells were immobilized on nickel grids and stained with 1% uranyl acetate prior to observation using an electron microscope. To observe the effect of the *cysK1* deletion, cells of the *cysK1* mutant were initially cultured in the presence of lanthionine prior to several hours of growth in its absence before TEM. Unlike the parent cells, which displayed a typical rod-shaped phenotype, the *cysK1* mutant cells became bulged in the absence of lanthionine, and this bulging defect was rescued by the ectopic expression of *cysK1* (Fig. 2C). These results demonstrate that the *cysK1* mutant is auxotrophic for lanthionine.

Next, to verify the previous inference that CysK1 is a major H_2_S producer (18), we compared the levels of H_2_S produced by the parental and *cysK1* mutant strains using a standard bismuth assay, in which H_2_S reacts with bismuth chloride to generate bismuth sulfide that can be measured by the optical density at 405 nm (OD_405_) (10, 26). Aliquots of fusobacterial cultures grown in the presence (parent and *cysK1* mutant strains) or absence (parent strain) of lanthionine were monitored at various time points to assay for cell growth and H_2_S production. As shown in Fig. 2D, no significant defect in growth was observed in the parent and *cysK1* mutant strains in the presence of lanthionine, compared to that of the parent strain grown in the absence of this metabolite. To our surprise, the *cysK1* mutant strain produced H_2_S at a level similar to that of the parent strain with or without lanthionine during the entire course of experimentation, i.e., over 20 h of growth in culture (Fig. 2E). It is most noteworthy that H_2_S was produced at the highest level when cells reached late log phase and that this was true regardless of the presence or absence of *cysK1* (Fig. 2E). The results demonstrate that *cysK1* is dispensable for H_2_S production *in vivo*, implying that some other enzyme must be involved.

### MegL is a major H_2_S-producing enzyme in F. nucleatum.

**FIG 3:**
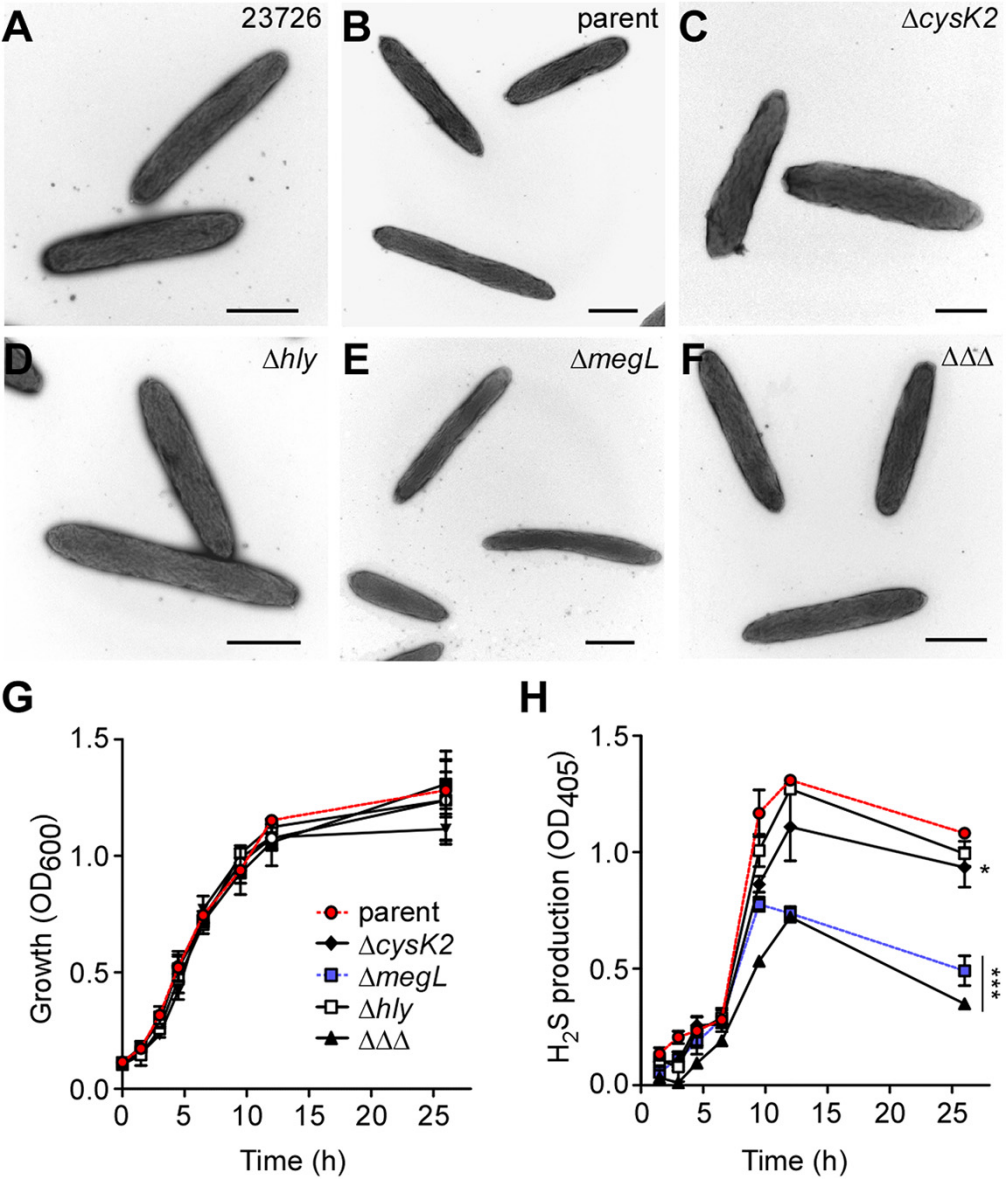
*megL* is largely required for H_2_S production in F. nucleatum. (A to F) Cells of the F. nucleatum parent strain or its isogenic mutant lacking *cysK2* (Δ*cysK2*), *megL* (Δ*megL*), *hly* (Δ*hly*), or all three genes (ΔΔΔ) were subjected to electron microscopy as described in the legend of Fig. 2C. Bars, 0.5 μm. (G and H) Growth and H_2_S production of the indicated F. nucleatum strains were determined as described in the legends of Fig. 2D and E, respectively.

To determine the role of the other three H_2_S-associated genes in H_2_S production *in vivo*, we generated nonpolar, in-frame deletion mutant strains that lack *cysK2* (Δ*cysK2*), *hly* (Δ*hly*), or *megL* (Δ*megL*) and a strain that lacked all three genes (ΔΔΔ), each of which was viable. These strains were then assayed for cell morphology, growth, and H_2_S production as mentioned above. TEM showed no noticeable morphological defects among mutant strains compared to the parent or the Δ*cysK1* mutant strain (Fig. 2C and Fig. 3A to F). Consistent with this result, each mutant grew at a rate comparable to that of the parent strain, with no apparent cell growth defect that could be detected (Fig. 3G).

We next examined H_2_S production in these strains over time using the bismuth assay mentioned above. Compared to the parental strain, the Δ*cysK2* and Δ*hly* mutants displayed a slight reduction in H_2_S production, whereas the Δ*megL* mutant exhibited a marked defect (Fig. 3H). The deletion of all three of the genes *cysK2*, *hly*, and *megL* further reduced H_2_S production, especially when cells were in the late stationary phase (Fig. 3H). To further confirm that the defect in H_2_S production observed in the Δ*megL* mutant is due to the lack of *megL* itself, we introduced into this mutant a plasmid that expressed *megL* from its native promoter. As expected, the ectopic expression of *megL* fully restored MegL expression, determined by immunoblotting using specific antibodies against MegL (see Fig. S1A in the supplemental material) and H_2_S production (Fig. S1B) in the Δ*megL* mutant.

10.1128/mbio.01936-22.1 FIG S1 Complementation of MegL rescues the defect of H_2_S production in the Δ*megL* mutant. (A) Whole-cell lysates of the parent, the *megL* mutant, and its rescued strain were analyzed by immunoblotting with anti-MegL. Molecular weight markers (in kilodaltons) are shown. (B) H_2_S production in the indicated strains was determined by the bismuth method with the absorbance measured at 405 nm. Download FIG S1, PDF file, 0.1 MB. Copyright © 2022 Chen et al. 2022 Chen et al. https://creativecommons.org/licenses/by/4.0/ This content is distributed under the terms of the Creative Commons Attribution 4.0 International license.

Since *megL* is predicted to be part of a transcriptional unit together with a downstream gene, *metT*, coding for a methionine transporter (22), according to the Genome Database Collection (https://biocyc.org), we sought to determine if *megL* and *metT* might be cotranscribed and functionally related. We first determined if *megL* or *metT* contains its own promoter by junction PCR using specific primers that target the 5′ untranslated region of *megL* (J1) or the intergenic region between *megL* and *metT* (J2) from cDNA (Fig. S2A). As shown in Fig. S2B, the upstream and downstream primer pair targeting the J1 junction in this assay amplified a predicted 300-bp product with genomic DNA (gDNA) only but not cDNA, whereas the other primer pair targeting the J2 junction amplified a predicted 400-bp product from both gDNA and cDNA templates. These results indicate that *megL*, and not *metT*, might contain a transcriptional promoter. To examine if this is the case, we performed 5′ RNA ligase-mediated rapid amplification of cDNA ends (5′ RLM-RACE) to locate the transcription start sites of both genes. Our results revealed two transcription start sites, both “A” at positions −32 and −34 upstream of *megL* (Fig. S2C), demonstrating that *megL* and *metT* are cotranscribed and that their expression is driven by a promoter upstream of *megL*.

10.1128/mbio.01936-22.2 FIG S2 *metT* is not required for H_2_S production. (A) *megL* and *metT* are predicted to be in the same transcriptional unit (https://biocyc.org). J1 and J2 are regions upstream of *megL* and between *megL* and *metT*, respectively. (B) The cotranscription of *megL* and *metT* was analyzed by RT-PCR with primer sets targeting J1 and J2 junctions. (C) A transcriptional unit of the *megL* locus was analyzed by 5′ RNA ligase-mediated rapid amplification of cDNA ends (5′ RLM-RACE). (D) Whole-cell lysates of the parent, *megL*, and *metT* strains were analyzed by immunoblotting with anti-MegL. A nonspecific band was used as a loading control. (E) H_2_S production in the indicated strains was determined by the bismuth method with the absorbance measured at 405 nm. Download FIG S2, PDF file, 0.2 MB. Copyright © 2022 Chen et al. 2022 Chen et al. https://creativecommons.org/licenses/by/4.0/ This content is distributed under the terms of the Creative Commons Attribution 4.0 International license.

We further determined if the deletion of *metT* affects *megL* expression and MegL-associated H_2_S production by generating a nonpolar, in-frame deletion of *metT*. As shown in Fig. S2D and E, there were no significant defects in MegL expression, as determined by immunoblotting with anti-MegL, and H_2_S production that could be observed for the Δ*metT* mutant. Altogether, these results reveal that MegL is the major H_2_S-producing enzyme of F. nucleatum *in vivo*.

### Expression of *megL* is modulated by the two-component signal transduction system ModSR.

The growth-phase-dependent induction of *megL*, as shown in Fig. 1, indicates that *megL* transcription is subject to some form of positive-negative regulation. This is consistent with our previously reported observation that *megL* is one of the numerous genes whose expression is upregulated in the absence of *modR*, the response regulator of a two-component signal transduction system (TCS), ModSR, as revealed by transcriptome sequencing (RNA-seq) (27). To confirm this result, here, we isolated total RNA samples from the parent, Δ*modR*, Δ*modS*, and complemented strains and measured the levels of the *megL* transcript in these strains by qRT-PCR. Of note, no significant changes in the expression levels of *cysk1*, *cysk2*, and *hly* were observed in the Δ*modR* mutant, relative to the parent strain, based on our previous RNA-seq analysis (27).

**FIG 4:**
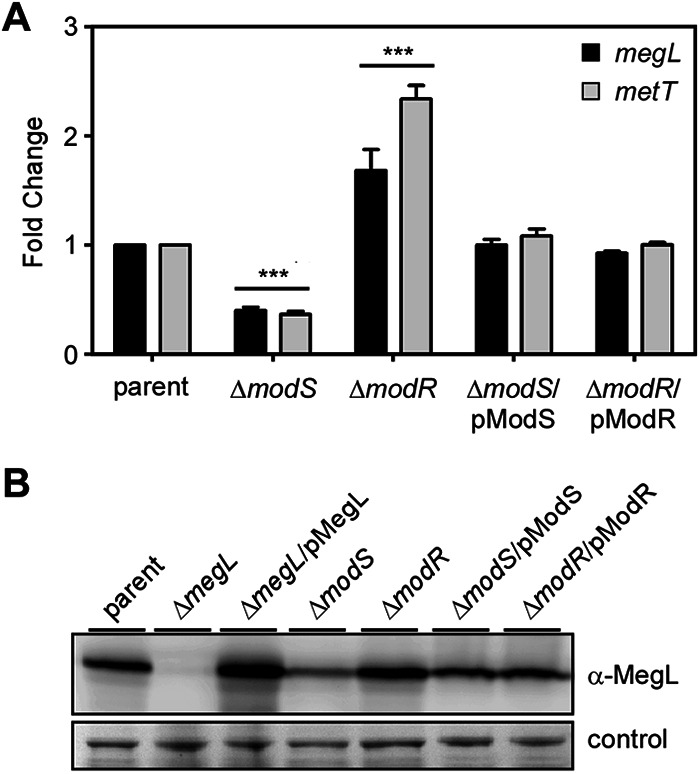
Expression of *megL* is modulated by the two-component system ModSR. (A) Cells of the indicated F. nucleatum strains grown to mid-log phase were harvested for total RNA isolation. The expression levels of *megL* and *metT* in the *modS* and *modR* mutants and their rescued strains, relative to the parent, were determined by qRT-PCR, with *rpoD* used as the internal control. (B) In a parallel experiment, whole-cell lysates of the indicated strains were obtained for immunoblotting with antibodies against MegL (α-MegL), with a nonspecific band stained with Coomassie blue used as a loading control.

As shown in Fig. 4, the transcription of *megL* was significantly decreased in a mutant devoid of *modS*, coding for the sensor kinase of the TCS ModSR, compared to the parent strain. In contrast, *megL* expression was highly increased in the *modR* mutant. This differential expression of *megL* seen in the two mutants was restored to the parental level with the ectopic expression of *modS* or *modR* in the respective mutants (Fig. 4A). In line with the results described above showing that *megL* and *metT* are in the same transcriptional unit (Fig. S2), the expression of *metT* mirrored that of *megL* in the parent strain, its isogenic *modS* and *modR* mutants, and their complemented strains (Fig. 4A).

To corroborate the RNA results reported above, we next analyzed the expression of MegL by immunoblotting samples from whole-cell lysates of these strains with anti-MegL. Consistent with the gene transcription results, MegL protein expression in the *modS* mutant was significantly reduced compared to that of the parent strain, and highly elevated expression of the MegL protein was observed in the *modR* mutant (Fig. 4B). Clearly, *megL* expression is modulated by the TCS ModSR: while ModR acts to repress MegL, ModS is required for MegL expression.

### MegL contributes to differential antibiotic sensitivity and tolerance.

**FIG 5:**
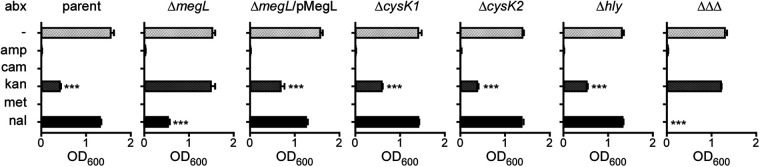
MegL contributes to the antibiotic susceptibility of F. nucleatum. Cells of the indicated strains grown anaerobically in TSPC containing ampicillin (amp) (100 μg/mL), chloramphenicol (cam) (50 μg/mL), kanamycin (kan) (50 μg/mL), metronidazole (met) (1 μg/mL), or nalidixic acid (nal) (25 μg/mL) for 24 h and the optical density at 600 nm (OD_600_) was taken to measure cell growth.

Previous studies in several model aerobic bacteria (Bacillus anthracis, Pseudomonas aeruginosa, Staphylococcus aureus, and Escherichia coli) revealed that H_2_S and H_2_S production-associated genes contribute to antibiotic tolerance (8, 28). To examine whether this is the case for F. nucleatum, we examined the susceptibility of the above-mentioned mutants to representative antibiotics of different classes. In a cell growth assay, fusobacterial strains cultured in liquid medium containing cysteine (TSPC [tryptic soy broth supplemented with 1% Bacto peptone plus 0.25% freshly made cysteine]) were treated with individual antibiotics for 24 h, and cell growth was determined by optical density (OD_600_) measurements (Fig. S3). The parent strain was highly sensitive to ampicillin (Amp), chloramphenicol (Cam), and metronidazole (Met) but somewhat tolerant to kanamycin (Kan) and resistant to nalidixic acid (Nal) (Fig. 5). Interestingly, our results revealed that while the Δ*cysK1*, Δ*cysK2*, and Δ*hly* mutants did not exhibit any deviation from sensitivity or tolerance to these antibiotics compared to the parent strain, the Δ*megL* mutant showed a measurable difference: it was resistant to Kan, and it also displayed some sensitivity to Nal (Fig. 5). These observed defects in the Δ*megL* mutant were rescued by the MegL-expressing plasmid. Strikingly, the triple mutant is completely sensitive to nalidixic acid (a DNA gyrase inhibitor). Taken together, these results support the notion that H_2_S and MegL positively contribute to the tolerance and sensitivity of fusobacteria to some but not all antibiotics that were tested.

10.1128/mbio.01936-22.3 FIG S3 MegL contributes to antibiotic tolerance in F. nucleatum. Cells of the indicated strains were anaerobically grown in TSPC containing ampicillin (amp) (100 μg/mL), chloramphenicol (cam) (50 μg/mL), kanamycin (kan) (50 μg/mL), metronidazole (met) (1 μg/mL), or nalidixic acid (nal) (25 μg/mL) for 24 h. The optical density at 600 nm (OD_600_) was taken to measure cell growth. Download FIG S3, PDF file, 2.6 MB. Copyright © 2022 Chen et al. 2022 Chen et al. https://creativecommons.org/licenses/by/4.0/ This content is distributed under the terms of the Creative Commons Attribution 4.0 International license.

### The *megL* mutant is attenuated in virulence in a mouse model of preterm birth.

**FIG 6:**
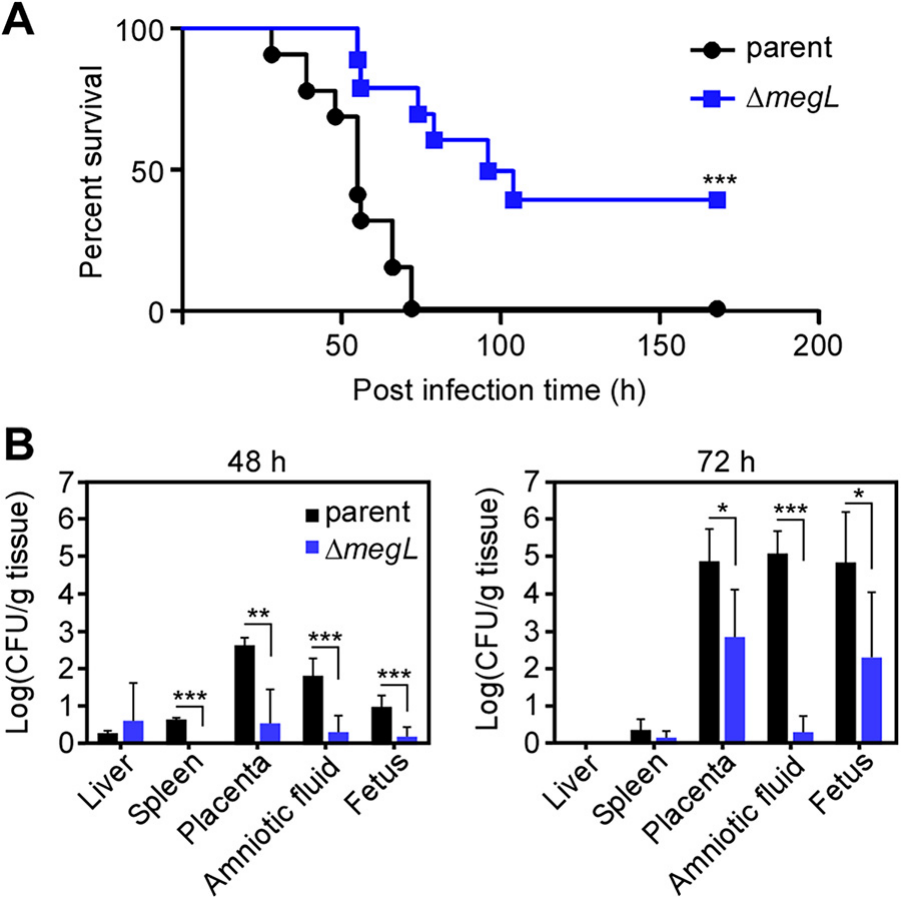
The *megL* mutant is attenuated in a murine model of preterm birth. (A) Groups of 5 pregnant CF-1 mice were injected via the tail vein with ~5 × 10^7^ CFU of the F. nucleatum parent strain (CW1) or its isogenic *megL* mutant on day 16 or 17 of gestation. Pup survival was monitored for the next 7 days. Significance was analyzed by the Mantel-Cox test using GraphPad (***, *P < *0.001). (B) Groups of 3 pregnant mice (see panel A) were used in the colonization experiment. At 48 and 72 h postinfection, the liver, spleen, placenta, amniotic fluid, and fetus from individual mice were harvested and homogenized for bacterial enumeration (CFU). Significance was analyzed by two-way analysis of variance (*, *P < *0.05; **, *P < *0.01; ***, *P < *0.001).

Since H_2_S production has been implicated in the virulence potential of some oral pathogens such as P. gingivalis (29), and MegL catabolizes l-cysteine to generate H_2_S and pyruvate (a key intermediate involved in many metabolic pathways), we examined whether the lack of *megL* affects fusobacterial virulence. We compared the infectivity of the Δ*megL* mutant with that of the parental strain using a mouse model of preterm birth previously developed for fusobacteria (22, 30). In this study, groups of 5 pregnant CF-1 mice on day 16/17 of gestation were intravenously injected with ~5 × 10^7^ CFU each of several bacterial strains to be tested, and pup survival was monitored over time. Strikingly, compared to almost no pup survival from mother mice challenged with the parent strain, nearly 40% pup survival was observed for mother mice challenged with the Δ*megL* mutant (Fig. 6A), thus revealing that MegL is likely a key virulence factor of F. nucleatum in causing adverse pregnancy outcomes.

To determine how the virulence attenuation phenotype observed for the Δ*megL* mutant correlates with the bacterial burdens in various critical organs, an infection experiment similar to the one described above was carried out, except that at 48 and 72 h postinfection, the liver, spleen, placenta, and amniotic fluid of mother mice, as well as their fetus, were harvested for bacterial enumeration (see Materials and Methods). Within 48 h postinfection, significant numbers of fusobacterial cells of the parental strain were detected in different organs, with the placenta and amniotic fluid containing higher bacterial loads than those in the other organs. In contrast, the fusobacterial loads were significantly reduced in the fetus and organs of animals infected by the Δ*megL* mutant (Fig. 6B). At 72 h postinfection, while bacterial cell counts of the parental strain were increasing in the placenta, amniotic fluid, and fetus of animals infected with this strain, the bacterial loads in similar organs of animals infected with the Δ*megL* mutant were significantly reduced (Fig. 6B). We conclude that MegL and H_2_S significantly contribute to placental colonization and fusobacterial virulence.

## DISCUSSION

H_2_S, a major volatile sulfur compound that significantly contributes to oral malodor or halitosis, is produced by many Gram-negative bacteria, of which F. nucleatum is known to be a high-H_2_S producer via cysteine metabolism (10, 31). Four enzymes that metabolize cysteine to produce H_2_S have been identified in certain F. nucleatum strains, including ATCC 25585: two cysteine synthases, CysK1 (FN1220) and CysK2 (FN1055); the l-cysteine desulfhydrase Hly (FN0625); and the l-methionine γ-lyase MegL (FN1419) (15–18). CysK1 has been considered a major H_2_S-producing enzyme in F. nucleatum as it exhibits high enzymatic activities *in vitro* that are correlated with high gene expression levels at the early log phase of growth (18). Nonetheless, the genetic and physiological determinants for these H_2_S biosynthetic enzymes remained to be determined. Here, by a combination of reverse genetics, biochemical methods, and rodent models of infection, we demonstrate that MegL not only is a major H_2_S-producing enzyme but also contributes significantly to the virulence and antibiotic susceptibility of F. nucleatum.

The finding that MegL is the major H_2_S-producing enzyme *in vivo* is supported by several lines of evidence. First, compared to the other three H_2_S-associated genes, *megL* is by far the most abundantly expressed gene throughout exponential growth, and its expression peaks at late exponential phase, concomitant with the highest level of H_2_S produced by the cells (Fig. 1 and 3). Second, the deletion of *megL* severely affects H_2_S production, whereas the deletion of *cysK2* has only marginal defects (Fig. 3). It is noteworthy that FN1419 in strain ATCC 25586 (or MegL in strain ATCC 23726) can catalyze γ-elimination reactions of l-methionine to produce methyl mercaptan, and *in vitro*, it was shown to have a low *k*_cat_ for H_2_S production from l-cysteine, leading to speculation that FN1419 might not be a major enzyme for cysteine metabolism (18). Third, although *cysK1* is highly expressed, albeit at levels significantly lower than those of *megL*, it is dispensable for H_2_S production (Fig. 2). A potential caveat is that the *cysK1* mutant cells are viable only in the presence of lanthionine (Fig. 2), which might interfere with cysteine metabolism that produces H_2_S. This appears not to be the case, as no significant difference in H_2_S production was observed in the parent strain in the presence and absence of lanthionine (Fig. 2E), although the parent cells seem to grow better in the presence of this compound than in its absence (Fig. 2D). It is worth mentioning that FN1220 (or CysK1) is considered a lanthionine synthase, possessing a unique structural loop involved in hydrogen bond contact with the product lanthionine, hence favoring it as an enzyme for H_2_S and l-lanthionine biosynthesis (32). Our finding that the *cysK1* mutant is a lanthionine auxotroph lends critical support for CysK1 as a lanthionine synthase in F. nucleatum. As such, CysK1 may not be a major H_2_S-producing enzyme in this organism.

A major finding emerging from this study establishes the roles of H_2_S and MegL in fusobacterial tolerance and susceptibility to certain antibiotics and the pathogenesis of fusobacteria in inducing adverse pregnancy outcomes (Fig. 5 and 6). In agreement with previous studies with several bacterial genera that suggested a general role of H_2_S in antibiotic tolerance (8), the *megL* mutant gained significant sensitivity to nalidixic acid (Fig. 5). Confoundingly, however, the *megL* mutant, which is substantially deficient in H_2_S production, has also gained resistance to kanamycin. This striking and opposing phenotypic change in antibiotic susceptibility resulting from defective H_2_S biosynthesis questions the idea of the universality of a mechanism in bacteria that enables H_2_S to confer tolerance to antibiotics. Previously, H_2_S has been suggested to protect bacteria from antibiotics by counteracting free radical species generated by antibiotics (8). Notably, the MegL-mediated metabolism of cysteine and methionine in the H_2_S biosynthesis pathway generates some key intermediates of energy metabolism (pyruvate) and a nitrogen source (ammonia) (Fig. 1 and 3). A simple explanation for the observed phenotypic differences in fusobacteria may be that a deficiency in the by-products of cysteine and methionine metabolism in the *megL* mutant reduces the ability of fusobacteria to combat free radicals imposed by antibiotics, which has been demonstrated for several aerobic bacteria that utilize a different set of enzymes to produce H_2_S (33). Similarly, reduced metabolic fitness caused by a deficiency in these metabolites could result in the attenuated virulence of the *megL* mutant due to its poor survival under the nutrient-deprived conditions of host tissues. Indeed, *megL* mutant cells are not well recovered in various tissues in infected pregnant mice, including the placenta, amniotic fluid, and fetus, compared to the parent cells (Fig. 6B). Finally, it is logical to imagine that the H_2_S-mediated S-persulfidation of multiple protein targets plays critical roles in the differential susceptibility of fusobacteria to a variety of antibiotics and their virulence in humans. Many additional genetic and phenotypic characterizations combined with transcriptomic and proteomic analyses and biochemical studies will be necessary to decipher these important issues of H_2_S physiology and genetics in fusobacteria. Given that *megL* homologs are present in many oral pathogens, including P. gingivalis, P. micra, and Treponema denticola, and that H_2_S itself is a diffusible metabolite that can be transmitted across species in the oral microbiome, F. nucleatum should provide an excellent experimental model to study MegL-mediated metabolism and bacterial virulence.

## MATERIALS AND METHODS

### Bacterial strains, plasmids, media, and growth.

All bacterial strains and plasmids used in this study are listed in Table S1 in the supplemental material. F. nucleatum strains were routinely cultured in Columbia broth or TSPC medium (tryptic soy broth supplemented with 1% Bacto peptone plus 0.25% freshly made cysteine) under anaerobic conditions (2% H_2_, 5% CO_2_, and 93% N_2_) at 37°C. E. coli strains were grown in Luria broth (LB) at 37°C. When necessary, media were supplemented with l-lanthionine at 1.5 mM, thiamphenicol at 5 μg/mL, or chloramphenicol at 15 μg/mL.

10.1128/mbio.01936-22.4 TABLE S1 Bacterial strains and plasmids used in this study. Download Table S1, PDF file, 0.2 MB. Copyright © 2022 Chen et al. 2022 Chen et al. https://creativecommons.org/licenses/by/4.0/ This content is distributed under the terms of the Creative Commons Attribution 4.0 International license.

Bacterial growth was monitored according to a previously reported protocol (27). Briefly, cultures of various F. nucleatum strains grown overnight were used to inoculate fresh cultures in Columbia broth with a starting OD_600_ of ~0.1. Bacterial growth at 37°C in an anaerobic chamber was monitored by OD_600_ measurements taken at timed intervals. The results presented are the averages from 3 independent experiments with duplicate measurements at each time point. Statistical analysis was performed with GraphPad Prism 5.0 (GraphPad, La Jolla, CA).

### Generation of gene deletion mutants in F. nucleatum.

Nonpolar, in-frame deletion mutants were generated using a GalK-based counterselection method described previously (20–22). The 1-kb flanking regions upstream and downstream of the gene of interest were cloned into the deletion vector pCM-GalK (34). The resulting plasmid was electroporated into the F. nucleatum CW1 strain (Table S1), and cointegrants resulting from a single-crossover event were selected on Columbia agar plates containing 5 μg/mL thiamphenicol. A single colony of cointegrants was inoculated into fresh Columbia broth without antibiotics to induce double-crossover homologous recombination, resulting in plasmid excision and generating wild-type and mutant alleles, which were selected on 0.25% 2-deoxygalactose (2-DG)-containing Columbia agar plates. Colony PCR was employed to screen for clones that harbor mutant alleles. To generate a triple mutant lacking *cysK2* (HMPREF0397_RS09315), *hly* (HMPREF0397_RS08940), and *megL* (HMPREF0397_RS04485), a single mutant was used as a starting strain.

### Plasmid construction. (i) pCysK1.

A DNA fragment containing the *cysK1* (HMPREF0397_RS07615) coding sequence and its promoter region was PCR amplified using primers pCysK1-KpnI and pCysK1-XhoI (Table S2) from chromosomal DNA of strain ATCC 23726. The amplified DNA fragment was digested with KpnI and XhoI and cloned into pCWU6 (Table S1) precut with the same restriction enzymes. The resulting plasmid was introduced into E. coli DH5α, and plasmid DNA was isolated for confirmation by colony PCR, restriction enzyme digestion, and DNA sequencing prior to electroporation into F. nucleatum.

10.1128/mbio.01936-22.5 TABLE S2 Primers used in this study. Download Table S2, PDF file, 0.1 MB. Copyright © 2022 Chen et al. 2022 Chen et al. https://creativecommons.org/licenses/by/4.0/ This content is distributed under the terms of the Creative Commons Attribution 4.0 International license.

### (ii) pMegL.

A DNA fragment containing the *megL* coding sequence and its promoter region was PCR amplified using primers pMegL-KpnI and pMegL-XhoI, using chromosomal DNA from strain ATCC 23726 as the template, and cloned into the KpnI and XhoI sites of plasmid pCWU6. The resulting plasmid was introduced into E. coli DH5α, and plasmid DNA was isolated for confirmation by colony PCR, restriction enzyme digestion, and DNA sequencing prior to electroporation into F. nucleatum.

### Detection of H_2_S.

Cultures of individual F. nucleatum strains grown overnight were used to inoculate fresh cultures into Columbia broth with a starting OD_600_ of ~0.1 in an anaerobic chamber at 37°C. At timed intervals, 400-μL aliquots were taken and mixed with the same volume of bismuth buffer [0.4 M triethanolamine-HCl (pH 8.0), 10 mM bismuth(III)chloride, 20 μM pyridoxal 5-phosphate monohydrate, 20 mM EDTA, and 40 mM l-cysteine] (10, 26). After 30 min of incubation at 37°C, H_2_S production was measured by the absorbance at 405 nm. The results presented are from 3 independent experiments performed in duplicate. Statistical significance was analyzed with GraphPad Prism 5.0 (GraphPad, La Jolla, CA).

### Quantitative reverse transcription-PCR and junction PCR.

RNA extraction was performed as previously described (21, 22). Cultures of various F. nucleatum strains grown overnight were used to inoculate fresh cultures in Columbia broth with a starting OD_600_ of ~0.1. At timed intervals, the total RNA of individual F. nucleatum strains was purified using RNeasy minikits (Qiagen) according to the manufacturer’s instructions, and contaminating DNA was removed by digestion with RNase-free DNase (Qiagen). Three hundred nanograms of RNA was used to synthesize cDNA using random hexadeoxyribonucleotide primers (Invitrogen) and Moloney murine leukemia virus (MMLV) reverse transcriptase (Invitrogen) according to the manufacturer’s protocol. Diluted cDNA samples were used for qRT-PCR with specific primer sets (Table S2), using iTaq SYBR green supermix (Bio-Rad) in a CFX96 Touch real-time PCR detection system (Bio-Rad). Data were obtained from three independent experiments, with *rpoD* or the 16S rRNA gene used as the control.

For junction PCR, approximately 1 μg pure RNA was used for cDNA synthesis with iScript reverse transcription supermix (Bio-Rad). A set of primers amplifying a region upstream of *megL* (J1) or a *megL* and *metT* junction (J2) was employed for junction PCR with gDNA or cDNA as the template.

### RNA ligase-mediated rapid amplification of cDNA ends.

5′ RNA ligase-mediated rapid amplification of cDNA ends (RLM-RACE) was performed using GeneRacer kits (Invitrogen) according to a previously reported protocol, with some modifications (35). First, ~4 μg of pure RNA was sequentially treated with shrimp alkaline phosphatase (New England BioLabs [NEB]) and RNA 5′ pyrophosphohydrolase (NEB). Treated RNA was ligated to an RNA oligonucleotide adapter using T4 RNA ligase I (NEB). All interstep purifications were done with phenol-chloroform extraction. The ligated RNA was then reverse transcribed using iScript RT supermix (Bio-Rad). The cDNA obtained was amplified with primer set RACE-F/MegL-R1 or RACE-F/metT-R3 using Phusion HF enzymes (Thermo Scientific).

### Western blotting.

For immunoblotting, a polyclonal antibody against recombinant MegL was generated according to a previously reported protocol (22). Briefly, primers LIC-megL-5 and LIC-megL-3 (Table S2) were used to PCR amplify the coding region of *megL*. The generated PCR product was cloned into the expression vector pMCSG7 (36). The recombinant plasmid, verified by DNA sequencing, was introduced into E. coli BL21(DE3) to produce the recombinant protein MegL with a His tag for protein purification by affinity chromatography. The purified protein was used for antibody production (Cocalico Biologicals, Inc.).

To detect MegL in fusobacteria, fusobacterial cells of various strains grown anaerobically at 37°C to mid- to late log phase (OD_600_ of ~1.0) were harvested by centrifugation. Cell pellets were suspended in 100 μL of sample buffer containing sodium dodecyl sulfate (SDS) and boiled for 10 min. Twenty-microliter aliquots were analyzed by SDS-PAGE using 12% polyacrylamide gels and immunoblotting with antibodies against MegL (1:5,000 dilution).

### Electron microscopy.

Transmission electron microscopy was performed according to previously reported protocols (21, 22). Briefly, fusobacteria grown in Columbia broth were harvested by centrifugation and suspended in 0.1 M NaCl. A drop of the bacterial suspension in phosphate-buffered saline was placed onto carbon-coated nickel grids and stained with 1% uranyl acetate. Samples were washed 5 times with water prior to imaging with an electron microscope.

### Antibiotic susceptibility assay.

An antibiotic susceptibility assay was performed based on previously reported methods, with some modifications (8, 28). Briefly, 1-mL aliquots of cultures of individual F. nucleatum strains in TSPC containing ampicillin (Amp) (100 μg/mL), chloramphenicol (Cam) (50 μg/mL), kanamycin (Kan) (50 μg/mL), metronidazole (Met) (1 μg/mL), or nalidixic acid (Nal) (25 μg/mL) were prepared with approximately 5 × 10^5^ CFU/mL of log-phase bacteria. Bacterial growth was monitored by the OD_600_ after 24 h of incubation at 37°C in an anaerobic chamber.

### Bacterial infection in mouse models.

A mouse model of preterm birth was performed according to previously described procedures (20, 22). Briefly, groups of five CF-1 (Charles River Laboratories) pregnant mice were infected via tail vein injection with ~5 × 10^7^ CFU of the F. nucleatum parent or *megL* mutant strain on day 16 or 17 of gestation. The numbers of live and dead pups were recorded during the next 7 days. Reproducibility was determined, statistical analysis was carried out relative to the parental strain, and significance was determined via the Mantel-Cox test, using GraphPad Prism 5.0.

For bacterial colonization, the same procedure as the one described above was performed; however, animals were sacrificed at 48 or 72 h postinjection. The liver, spleen, placenta, amniotic fluid, and fetus were harvested, weighed, and homogenized for bacterial enumeration. Three pregnant mice per group were used for every time point, and reproducibility was determined. Statistical analysis was carried out relative to the parental strain, and significance was determined via Student’s *t* test, using GraphPad Prism 5.0. Statistical significance was set at a *P* value of <0.05. (***, *P < *0.001; **, *P < *0.01; *, *P < *0.05). All animal procedures were approved by the UCLA Animal Research Committee.
