# Supplementary figures and images for: Genetic Determinants of Hydrogen Sulfide Biosynthesis in Fusobacterium nucleatum Are Required for Bacterial Fitness, Antibiotic Sensitivity, and Virulence
Source: mBio. 2022 Sep 8;13(5):e01936-22. doi: 10.1128/mbio.01936-22 (PMC9600241; doi:10.1128/mbio.01936-22)

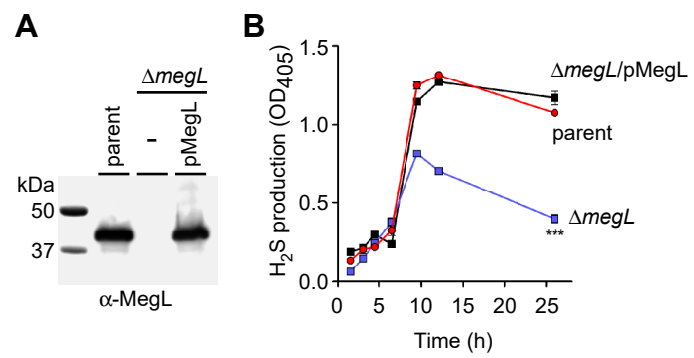

Figure S1: Chen et al.

Supplement: FIG S1 [file mbio.01936-22-s0001.pdf]

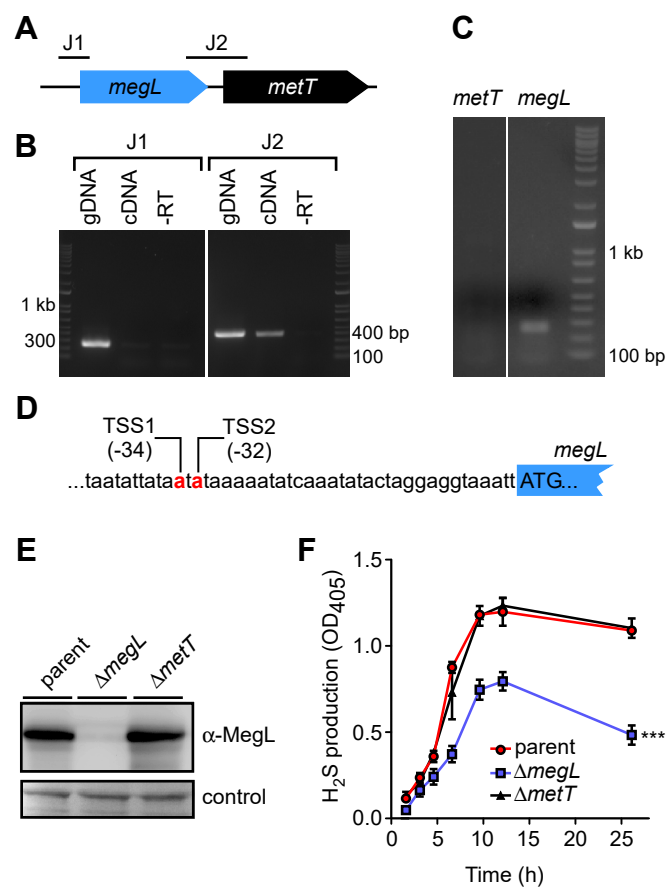

Figure S2: Chen et al.

Supplement: FIG S2 [file mbio.01936-22-s0002.pdf]

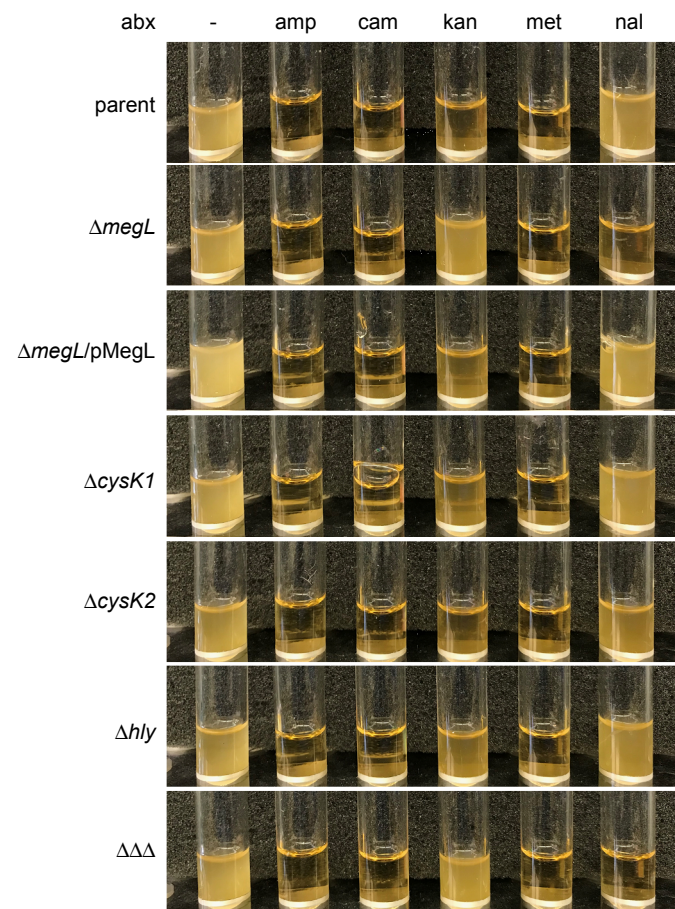

Figure S3: Chen et al.

Supplement: FIG S3 [file mbio.01936-22-s0003.pdf]
