# Supplementary material for: Genetic Determinants of Hydrogen Sulfide Biosynthesis in Fusobacterium nucleatum Are Required for Bacterial Fitness, Antibiotic Sensitivity, and Virulence
Source: mBio. 2022 Sep 8;13(5):e01936-22. doi: 10.1128/mbio.01936-22 (PMC9600241; doi:10.1128/mbio.01936-22)
Supplement: TABLE S1 [file mbio.01936-22-s0004.pdf]

Supplemental Tables

Supplemental Table S1: Bacteria strains and plasmids used in this study.

Strain and Plasmid	Description	Reference
Strain		
F. nucleatum ATCC 23726	Urogentical tract isolate	(1)
F. nucleatum CW1	Isogenic derivative of ATCC 23726; lacking *galK*	(1)
Δ*cysk1*	Isogenic derivative of CW1; lacking *cysK1*	This study
Δ*cysK2*	Isogenic derivative of CW1; lacking *cysK2*	This study
Δ*megL*	Isogenic derivative of CW1; lacking *megL*	This study
Δ*hly*	Isogenic derivative of CW1; lacking *hly*	This study
Δ*cysK2*Δ*megL*Δ*hly*	Isogenic derivative of CW1; lacking *cysk2*, *megL*, and *hly*	This study
Δ*cysk1*/pCysK1	CysK1 complementation strain	This study
Δ*megL*/pMegL	MegL complementation strain	This study
Δ*modS*	Isogenic derivative of CW1; lacking *modS*	(2)
Δ*modR*	Isogenic derivative of CW1; lacking *modR*	(2)
Δ*modS*/pModS	ModS complementation strain	(2)
Δ*modR*/pModR	ModR complementation strain	(2)
Plasmid		
pCWU6	Derivative of pHS30	(1)
pCM-galK	GalK-based vector for gene deletion	(3)
pΔ*cysk1*	Derivative of pCM-galK; deletion vector of *cysK1*	This study
pΔ*cysK2*	Derivative of pCM-galK; deletion vector of *cysK2*	This study
pΔ*megL*	Derivative of pCM-galK; deletion vector of *megL*	This study
pΔ*hly*	Derivative of pCM-galK; deletion vector of *hly*	This study
pCysk1	pCWU6 expressing CysK1	This study
pMegL	pCWU6 expressing MegL	This study
pMCSG7-MegL	pMCSG7 expressing H_6-MegL	This study

References

1. Wu C, Al Mamun AAM, Luong TT, Hu B, Gu J, Lee JH, D'Amore M, Das A, Ton-That H. 2018. Forward Genetic Dissection of Biofilm Development by *Fusobacterium nucleatum*: Novel Functions of Cell Division Proteins FtsX and EnvC. mBio 9.

2. Scheible M, Nguyen CT, Luong TT, Lee JH, Chen YW, Chang C, Wittchen M, Camacho MI, Tiner BL, Wu C, Tauch A, Das A, Ton-That H. 2022. The Fused Methionine Sulfoxide Reductase MsrAB Promotes Oxidative Stress Defense and Bacterial Virulence in *Fusobacterium nucleatum*. mBio 13:e0302221.

3. Nariya H, Miyata S, Suzuki M, Tamai E, Okabe A. 2011. Development and application of a method for counterselectable in-frame deletion in *Clostridium perfringens*. Appl Environ Microbiol 77:1375-82.
