# Supplementary material for: Genetic Determinants of Hydrogen Sulfide Biosynthesis in Fusobacterium nucleatum Are Required for Bacterial Fitness, Antibiotic Sensitivity, and Virulence
Source: mBio. 2022 Sep 8;13(5):e01936-22. doi: 10.1128/mbio.01936-22 (PMC9600241; doi:10.1128/mbio.01936-22)
Supplement: TABLE S2 [file mbio.01936-22-s0005.pdf]

Supplementary Table S2: Primers used in this study.

Primer	Sequence[a]	Application
cysK1-F-A-BamHI	CGCGGATCCCATTCTTTAAATGAAAAAGAATTAC	Δ*cysK1*
cysK1-R-A-PstI	AAAACTGCAGATTTAACAACATTGTAAAATTAACC	Δ*cysK1*
cysK1-F-B	TAAAAGTTTTAAGAAGTTGTTATAAATTTATAGCAAC	Δ*cysK1*
cysK1-R-B	CATAAAAATCCTCCAATCAAACTTTGTTTTC	Δ*cysK1*
cysK2-F-A-BamHI	CGCGGATCCCAAAATCTTCTTCTAAATTTCCAAATTTAAG	Δ*cysK2*
cysK2-R-A-PstI	AAAACTGCAGGTTCTTTATATCTCAACAGATAAAATTAATG	Δ*cysK2*
cysK2-F-B	TAAAATTATTAAAATCAAAAAAGGTTGTTGCAAATTAC	Δ*cysK2*
cysK2-R-B	CATTTTTAAGCCTCCATTATTATTTTTTAATAAAGC	Δ*cysK2*
megL-F-A-BamHI	CGCGGATCCGACATTCTCTTGAATTATAAAAAAAATCTG	Δ*megL*
megL-R-A-PstI	AAAACTGCAGCCATTATAGATTTCTTTCCCATAACC	Δ*megL*
megL-F-B	TAACTTTACTCATTTGTCTTAATTCCTTAC	Δ*megL*
megL-R-B	TTCCATAATTTACCTCCTAGTATATTTG	Δ*megL*
hly-F-A-BamHI	CGCGGATCCCAATGTGCTTATTAGAAGAAATGCAATG	Δ*hly*
hly-R-A-SacI	CCCGAGCTAGCATTTTTTCTACTTTTTATTTTTGATATTATAC	Δ*hly*
hly-F-B	TAATATGAAAAGTGAATTAAAAGAAAAAAAATATGG	Δ*hly*
hly-R-B	CATAATTTTCTCCTCTTATATATTAATTCTTTTG	Δ*hly*
pCysk1-F-KpnI	CGGGGTACCCTTCTCTTCTTCTTAGAAAAAAAATTTTAG	pCysk1
pCysK1-R-XhoI	CCGCTCGAGTTATAAATCACAAATATTAGATAAATATTTTTC G	pCysk1
pMegL-F-KpnI	CGGGGTACCGATTATATCCCTTTTTTACAAAACAAC	pMegL
pMegL-R-XhoI	CCGCTCGAGCCATATCCTATATTTTAAAGAACTAATTTATGT ACTTGTC	pMegL
LIC-megL-5	TACTTCCAATCCAATGCAATGGAAATGAAAAAATCTGGTTT AGG	pMCSG7-MegL
LIC-megL-3	TTATCCACTTCCAATGTTAAATTTTTTCTAGTCCTTGTTCTA AATC	pMCSG7-MegL
rpoD-F	AGGGAACATAGGCCTTATGAAAG	qRT-PCR
rpoD-R	GCCTGTCTTATCCACCAAGTAG	qRT-PCR
16S-F	GGTTAAGTCCCGCAACGA	qRT-PCR
16S-R	CATCCCCACCTTCCTCCTAC	qRT-PCR
cysK1-F	AACAGGGACAGGAGGTAGTT	qRT-PCR
cysK1-R	AGATGAAGCAGGCTCAACAG	qRT-PCR
cysk2-F	GCTACAAGTGGAAACACAGGA	qRT-PCR
cysK2-R	TCACTCATCCAATCTGGCATATAA	qRT-PCR
megL-F	CACAAGACTAGGCAATCCTACA	qRT-PCR
megL-R	GCTCCCATACCAGATGACATAG	qRT-PCR
hly-F	CTATGTGGGTCGCTGATATGG	qRT-PCR
hly-R	CACTGACATAAGAATATCCAAATACCC	qRT-PCR
metT-F	GGGAAAGTGCAGCTATTCTTGA	qRT-PCR
metT-R	GGAGCAACTTTATCTCCAGCAA	qRT-PCR
MegL-F1	CCATTTATTTCCTAACATGTCCTG	RT-PCR

MegL-F2	AGCTGATTTAGAACAAGGACTAG	RT-PCR
MegL-R1	CCTGCATGTATAGCAGTTGTTC	RT-PCR/ RACE
metT-R1	CTGGATGACCACAACCTTCAAG	RT-PCR
metT-R3	CCTTTAAAACTAGCCTTAGTCTCC	RACE
RACE-F	GACTGGAGCACGAGGACACT	RACE
RACE-nF	GACACTGACATGGACTGAAGGAG	RACE
RNA oligo	CGACUGGAGCACGAGGACACUGACAUGGACUGAAGGAG UAGAAA	RACE

[a] Engineered restriction sites are underlined.
